# An Improved Strong Tracking Cubature Kalman Filter for GPS/INS Integrated Navigation Systems

**DOI:** 10.3390/s18061919

**Published:** 2018-06-12

**Authors:** Kaiqiang Feng, Jie Li, Xi Zhang, Xiaoming Zhang, Chong Shen, Huiliang Cao, Yanyu Yang, Jun Liu

**Affiliations:** 1 Key Laboratory of Instrumentation Science & Dynamic Measurement, Ministry of Education, North University of China, Taiyuan 030051, China; B1506011@st.nuc.edu.cn (K.F.); Zhangxi@nuc.edu.cn (X.Z.); zxm_auto@nuc.edu.cn (X.Z.); shenchong@nuc.edu.cn (C.S.); caohuiliang@nuc.edu.cn (H.C.); S1506116@st.nuc.edu.cn (Y.Y.); liuj@nuc.edu.cn (J.L.); 2 National Key Laboratory for Electronic Measurement Technology, North University of China, Taiyuan 030051, China

**Keywords:** GPS/INS integrated navigation, cubature Kalman filter, strong tracking filter, spherical simplex-radial rule

## Abstract

The cubature Kalman filter (CKF) is widely used in the application of GPS/INS integrated navigation systems. However, its performance may decline in accuracy and even diverge in the presence of process uncertainties. To solve the problem, a new algorithm named improved strong tracking seventh-degree spherical simplex-radial cubature Kalman filter (IST-7thSSRCKF) is proposed in this paper. In the proposed algorithm, the effect of process uncertainty is mitigated by using the improved strong tracking Kalman filter technique, in which the hypothesis testing method is adopted to identify the process uncertainty and the prior state estimate covariance in the CKF is further modified online according to the change in vehicle dynamics. In addition, a new seventh-degree spherical simplex-radial rule is employed to further improve the estimation accuracy of the strong tracking cubature Kalman filter. In this way, the proposed comprehensive algorithm integrates the advantage of 7thSSRCKF’s high accuracy and strong tracking filter’s strong robustness against process uncertainties. The GPS/INS integrated navigation problem with significant dynamic model errors is utilized to validate the performance of proposed IST-7thSSRCKF. Results demonstrate that the improved strong tracking cubature Kalman filter can achieve higher accuracy than the existing CKF and ST-CKF, and is more robust for the GPS/INS integrated navigation system.

## 1. Introduction

GPS/INS integration navigation systems have drawn great attention due to their widespread applications in the areas of dynamic navigation and positioning [1,2,3]. Depending on the information fusion level, three main forms of integration structures have been developed: loosely coupled [4], tightly coupled [5] and deeply coupled [6]. In this paper, only the loosely coupled integrated navigation system is considered due to its easier implementation and appropriate performance. For GPS/INS integration, the most celebrated mechanism is the Kalman type filter and it has been widely used in the practical systems. In the Kalman type filter, the optimal estimation can be achieved when the distributions of both process noise and measurement noise are assumed to be Gaussian and the corresponding statistics are accurately known [7]. However, due to the influence of vehicle’s severe maneuvers in the actual applications, the Gaussian assumption may be violated and the statistics may be uncertain, which will result in a degraded performance of the conventional KF-based GPS/INS integration system [8,9]. Therefore, in the presence of process uncertainties, there is a great demand to develop an effective filter for better suppressing them, which is the main focus of this work.

To address the filtering problems with process uncertainty effectively, a large number of fruitful algorithms have been investigated, such as the particle filter (PF) [10], the variational Bayesian-based adaptive Kalman filter (VB-AKF) [11,12,13,14], the maximum correntropy-based Kalman filter (MC-KF) [15,16,17,18,19] and the Huber’s M-Estimation-based Kalman filter (HKF) [8]. The particle filter is based on the sequence Monte Carlo sampling technique and can solve the nonlinear non-Gaussian system state estimation problem effectively [10]. It is, however, difficult to use the particle filter to cope with the high dimensional filter problem since the computational complexity increases exponentially with the dimensions of the state. The variational Bayesian-based adaptive Kalman filter can estimate the statistics of the time-varying process noise variance online by approximating the joint posterior distribution of the latent state and noise variance by a factorized free form distribution. However, the dynamic model designed for variance parameters used in the VB-AKF is heuristic and relatively rough, which may degrade its estimation accuracy when the process noise covariance changes frequently [14]. The maximum correntropy-based Kalman filter is capable of suppressing the larger process uncertainty by maximizing the correntropy of the predicted error and residual. However, it is very difficult to determine the appropriate value of kernel bandwidth size in actual applications, which may degrade the estimation performance of the MC-KF [16]. The Huber’s M-Estimation-based Kalman filter handles the process uncertainty by employing the cost function of M-estimator to construct weighting matrix, which can rescale the prior state estimate covariance as a result the process uncertainty are suppressed. Unfortunately, the influence function of the HKF doesn’t redescend, which results in limited estimation accuracy [20].

Another way of solving the problems of process uncertainty is to use adaptive filters where the online estimates of process noise statistics are obtained together with the dynamic state. The adaptive filters fall into two categories, i.e., the Multiple Model Adaptive Kalman filter (MM-AKF) [21] and the Innovation-based Adaptive Kalman filter (IAKF) [22]. The multiple model adaptive Kalman filter handles the process uncertainty by running a bank of Kalman filters with different stochastic models in parallel. However, it is only suitable for systems with known dynamics since it operates under the assumption that one of the models in the model bank is the correct one [22,23]. The Innovation-based Adaptive Kalman filter can estimate the appropriate covariance matrix of process noise by forcing the innovation sequence of the Kalman filter to be white Gaussian noise sequence with zero mean. However, this algorithm suffers from the limitation that the convergence to the right process noise covariance matrix can’t be guaranteed and rather large windows of data are required to achieve a reliable estimation of process noise covariance matrix, making it only suitable for slowly changing systems [22].

A feasible approach to deal with the process uncertainty is to employ the strong tracking filter [24,25,26]. The principle of strong tracking filter is that the strong tracking factor can maintain the residual sequence orthogonal by introducing the time-variant suboptimal fading factor matrix to the state prediction covariance matrix. As a result, the strong tracking filter provides a strong robustness against process uncertainty. However, the conventional strong tracking filter may suffer from the following problems: (1) The suboptimal fading factor is incorporated in the whole filtering process, which may result in the loss of precision when there is no process uncertainty existed; (2) The symmetry of the prediction covariance matrix cannot be guaranteed when the suboptimal fading factor with different diagonal elements is carried out on the error covariance matrix, which may degrade the filtering performance and even lead to the divergence.

Thus, in order to solve the issues mentioned above, an improved strong tracking cubature Kalman filter that could be applied to solve the high dimensional GPS/INS integrated navigation problem is proposed in our paper. First, the hypothesis testing method is adopted to identify process model uncertainty, which avoids the loss of precision when there is no process uncertainty in the dynamic system. In addition, a defined suboptimal fading factor, which is based on the idea of Cholesky decomposition, is proposed to ensure the symmetry of error covariance matrix when the fading factor is introduced. Second, the seventh-degree spherical simplex-radial cubature rule, which is used to compute the more exact Gaussian type integral, is integrated into the strong tracking cubature Kalman filter to improve the estimation accuracy and numerical stability of the GPS/INS integrated navigation system. Finally, the car-mounted experiment is conducted to evaluate the effectiveness of the proposed approach. The experiment results indicate that the proposed IST-7thSSRCKF method has better robustness for the suppression of process uncertainties as compared with CKF and STCKF for the GPS/INS integrated navigation problem.

The reminder of this paper is organized as follows: the strong tracking filter and cubature Kalman filter are briefly reviewed in Section 2. Section 3 presents the improved STCKF for SINS/GPS integration. In Section 4, experiments are carried out to evaluate the performance of the proposed approach. Concluding remarks are given in Section 5.

## 2. The Strong Tracking Filter and Cubature Kalman Filter

In this section, the strong tracking filter and cubature Kalman filter are briefly reviewed and discussed respectively. Consider the following nonlinear discrete-time dynamic system with additive process and measurement noise:

(1) $$\left\{ \begin{array}{l} x_{k}=f\left(x_{k-1}\right)+w_{k-1}\\ z_{k}=h\left(x_{k}\right)+v_{k} \end{array}\right.$$

where $x_{k}\in {\mathbb{R}}^{n\times 1}$, $z_{k}\in {\mathbb{R}}^{m\times 1}$ are the state vector of the dynamic system and measurement vector at discrete time k; f(⋅) denotes the nonlinear dynamic model and h(⋅) represents the measurement model. $w_{k-1}$ and $v_{k}$ are assumed to be independent process and measurement Gaussian noise sequences with zero means and variances $Q_{k-1}$ and $R_{k}$, respectively.

### 2.1. Strong Tracking Filter

The strong tracking filter, proposed by Zhou et al. [27], is essentially a kind of Kalman filter which is based on the orthogonal principle of the output residual error. In the strong tracking filter, the time-varing suboptimal fading factor $\lambda_{k}$ is introduced to the predicted error covariance matrix $P_{k|k-1}$, which adjusts the gain matrix $K_{k}$ online in real time, making sure that the innovations remain orthogonal and the available information in the residuals $\varepsilon_{k}$ can be extracted completely. In this way, the algorithm has strong robustness against process uncertainty. The general structure of the strong tracking filter algorithm can be expressed as follows:

(2) $$\varepsilon_{k}=z_{k}-h({\hat{x}}_{k|k-1})$$

(3) $${\hat{x}}_{k|k}={\hat{x}}_{k|k-1}+K_{k}\varepsilon_{k}$$

where ${\hat{x}}_{k|k}$ is the estimate value at discrete time k, $\varepsilon_{k}$ denotes the residual sequence of measurement. The question of strong tracking filter is how to adjust the time-varing gain matrix online according to the measurement innovation. As we known, if there is no process uncertainty in the system model, the strong tracking filter boils down to the standard Kalman filter and the time-varing gain matrix $K_{k}$ can be determined directly as follows:

(4) $$K_{k}=P_{k|k-1}H_{k}^{T}{\left(H_{k}P_{k|k-1}H_{k}^{T}+R_{k}\right)}^{-1}$$

(5) $$P_{k|k-1}=F_{k|k-1}P_{k-1}F_{k|k-1}^{T}+Q_{k-1}$$

where $F_{k|k-1}={\frac{\partial f}{\partial x}|}_{x={\hat{x}}_{k-1}}$ and $H_{k}={\frac{\partial h}{\partial x}|}_{x={\hat{x}}_{k|k-1}}$ are the system transition matrix and observation matrix, respectively. However, the process uncertainty in actual application results in a deviation between the state estimation and actual state, leading to the nonorthogonality among the residuals. Actually, the nonorthogonality indicates that the available information in the measurement is not fully used in the filtering estimation. Thus, the suboptimal fading factor $\lambda_{k}$ is introduced to the state predicted error covariance matrix, which will deliberately decrease the effect of historical information in the process estimation and keep the residual sequence mutually orthogonal. The predicted error covariance matrix after incorporating the fading factor can be expressed as follows:

(6) $$P_{k|k-1}=\lambda_{k}F_{k|k-1}P_{k-1}F_{k|k-1}^{T}+Q_{k-1}$$

where the suboptimal fading factor $\lambda_{k}$ is determined by the orthogonal principle and a popular choice of this can be given by [28]:

(7) $$\lambda_{k}=\{\begin{array}{c} \alpha c_{k},\;\alpha c_{k}\geq 1\;\\ 1,\;\alpha c_{k}<1\; \end{array},c_{k}=\frac{tr\left[N_{k}\right]}{tr\left[M_{k}\right]}$$

where α(α≥1) denotes the proportional coefficient of fading factor $\lambda_{k}$, which is a constant and can be predetermined by a prior knowledge. $tr\left[N_{k}\right]$ and $tr\left[M_{k}\right]$ represent the trace of the matrix $N_{k}$ and $M_{k}$, respectively. $N_{k}$ and $M_{k}$ are defined as follows:

(8) $$N_{k}=V_{k}-H_{k}Q_{k-1}{Η}_{k}^{T}-\beta R_{k}$$

(9) $$M_{k}=H_{k}F_{k|k-1}P_{k-1}F_{k|k-1}^{T}H_{k}^{T}$$

(10) $$V_{k}=\{\begin{array}{c} \varepsilon_{1}\varepsilon_{1}^{T},\;k=0\\ \frac{\rho V_{k-1}+v_{k}v_{k}^{T}}{1+\rho },\;k\geq 1\; \end{array}$$

where $V_{k}$ denotes the residual error sequence covariance matrix. β denotes the softening factor of the residual sequence, which is used to improve the smoothness and accuracy of the state estimation, generally β=4.5 [28]. ρ is the fading factor satisfying 0.95≤ρ<0.995, usually, we set ρ to be 0.95 [29].

### 2.2. Cubature Kalman Filter

The cubature Kalman filter was first presented by Haykin to solve high-dimensional nonlinear filtering problems [30]. The heart of cubature Kalman filter is to use the third-degree cubature rule to approximate the integral of form (nonlinear function × Gaussian density) instead of approximating the nonlinear function. Based on the third-degree cubature rule, a set of 2n cubature points with the same weight are chosen to propagate the state and covariance matrix at each update cycle. The integral with respect to the general Gaussian weighted integral can be approximated by the cubature rule as follows:

(11) $$\begin{array}{l} I\left(f\right)=\int_{{\mathbb{R}}^{n}}f(x)\;N\left(x;\bar{x},P\right)dx=\int_{{\mathbb{R}}^{n}}f(\sqrt{P}x+\bar{x})\;N\left(x;0,I\right)dx\\ \approx \sum_{i=1}^{m}\omega_{i}f\left(\sqrt{P}\xi_{i}+\bar{x}\right) \end{array}$$

where $P=\sqrt{P}{\sqrt{P}}^{T}$;$\sqrt{P}$ can be calculated by the Cholesky decomposition or the singular value decomposition. f(x) is the arbitrary nonlinear function, ${\mathbb{R}}^{n}$ is the integral domain, $\bar{x}$ is the mean of x, *m* is the total number of cubature points. $\left\{\xi_{i},\omega_{i}\right\}$ are the ith cubature point and the corresponding weight, respectively, which are given by:

(12) $$\xi_{i}=\sqrt{\frac{m}{2}}{\left[1\right]}_{i}$$

(13) $$\omega_{i}=\frac{1}{m},i=1,2,\ldots m=2n$$

where ${\left[1\right]}_{i}$ denotes the ith column vector of the points set [1]. For example, $\left[1\right]\in {\mathbb{R}}^{2}$ represents the following set of points:

(14) $$\left\{\left(\begin{array}{c} 1\\ 0 \end{array}\right),\left(\begin{array}{c} 0\\ 1 \end{array}\right),\left(\begin{array}{c} -1\\ 0 \end{array}\right),\left(\begin{array}{c} 0\\ -1 \end{array}\right)\right\}$$

Under the assumption that the initial state ${\hat{x}}_{0|0}$ and initial error covariance matrix ${\hat{P}}_{0|0}$ are known, the computational steps of the cubature Kalman filter can be summarized as follows [30]:

#### 2.2.1. Prediction

(1) Factorize the covariance and evaluate the cubature points (i=1,2,…2n):
  (15) $$P_{k-1|k-1}=S_{k-1|k-1}S_{k-1|k-1}^{T}$$
  (16) $$X_{i,k-1|k-1}=S_{k-1|k-1}\xi_{i}+{\hat{x}}_{k-1|k-1}$$
(2) Evaluate the propagated cubature points through the process model:
  (17) $$X_{i,k|k-1}^{*}=f\left(X_{i,k-1|k-1}\right)$$
(3) Estimate the predicted state and the corresponding error covariance:
  (18) $${\hat{x}}_{k|k-1}=\frac{1}{2n}\sum_{i=1}^{2n}X_{i,k|k-1}^{*}$$
  (19) $$P_{k|k-1}=\frac{1}{2n}\sum_{i=1}^{2n}X_{i,k|k-1}^{*}X_{i,k|k-1}^{{*}^{T}}-{\hat{x}}_{k|k-1}{\hat{x}}_{k|k-1}^{T}+Q_{k-1}$$

#### 2.2.2. Update

(1) Factorize the covariance and evaluate the cubature points (i=1,2,…2n):
  (20) $$P_{k|k-1}=S_{k|k-1}S_{k|k-1}^{T}$$
  (21) $$X_{i,k|k-1}=S_{k|k-1}\xi_{i}+{\hat{x}}_{k|k-1}$$
(2) Evaluate the propagated cubature points through the observation model:
  (22) $$Z_{i,k|k-1}=h\left(X_{i,k|k-1}\right)$$
(3) Estimate the predicted measurement:
  (23) $${\hat{z}}_{k|k-1}=\frac{1}{2n}\sum_{i=1}^{2n}Z_{i,k|k-1}$$
(4) Estimate the covariance and Kalman gain:
  (24) $$P_{zz,k|k-1}=\frac{1}{2n}\sum_{i=1}^{2n}Z_{i,k|k-1}Z_{i,k|k-1}-{\hat{z}}_{k|k-1}{\hat{z}}_{k|k-1}^{T}+R_{k}$$
  (25) $$P_{xz,k|k-1}=\frac{1}{2n}\sum_{i=1}^{2n}X_{i,k|k-1}Z_{i,k|k-1}^{T}-{\hat{x}}_{k|k-1}{\hat{z}}_{k|k-1}^{T}$$
  (26) $$K_{k}=P_{xz,k|k-1}/P_{zz,k|k-1}$$
(5) Estimate the updated state and the corresponding error covariance:
  (27) $${\hat{x}}_{k|k}={\hat{x}}_{k|k-1}+K_{k}\left(z_{k}-{\hat{z}}_{k|k-1}\right)$$
  (28) $$P_{k|k}=P_{k|k-1}-K_{k}P_{zz,k|k-1}K_{k}^{T}$$

## 3. An Improved Strong Tracking 7thSSRCKF Algorithm

### 3.1. The Improvement of Strong Tracking Kalman Filter

In order to improve the tracking performance of the traditional strong tracking filtering algorithm for multi-variable nonlinear system, an improved time-varying fading factor is proposed in this section. In the proposed improved strong tracking Kalman filter, the hypothesis testing method is adopted to identify process model uncertainty, which avoids the loss of precision when there is no process uncertainty in the dynamic system. In addition, a defined suboptimal fading factor, which is based on the idea of least squares and Cholesky decomposition, is proposed to ensure the symmetry of error covariance matrix when the fading factor is introduced.

#### 3.1.1. Process Uncertainty Identification

For the dynamic system without process uncertainty, the innovation $\varepsilon_{k}$ should be zero-mean Gaussian-distribution with covariance $H_{k}P_{k|k-1}H_{k}^{T}+R_{k}$ when the filtering is stable [31]. So the square of the Mahalanobis distance of the innovation should obey a $\chi^{2}$ distribution [32], then we define the statistical information of predicted residual vector as follows:

(29) $$\gamma_{k}=\varepsilon_{k}^{T}{\left[H_{k}\left(F_{k|k-1}P_{k-1}F_{k|k-1}^{T}+Q_{k-1}\right)H_{k}^{T}+R_{k}\right]}^{-1}\varepsilon_{k}∼\chi^{2}\left(m\right)$$

where $\gamma_{k}$ obeys a Chi-square distribution with *m* degrees of freedom, m is the number of state variable that can be observed directly. According to the hypothesis testing theory, for a chosen significant level α, we have:

(30) $$P\left(\chi^{2}<\chi_{\alpha ,m}^{2}\right)=1-\alpha$$

where the α-quantile $\chi_{\alpha ,m}^{2}$ of the Chi-square distribution is predetermined. In this paper, the value is set $\alpha =0.05,\;\chi_{\alpha ,m}^{2}=12.592$. If the actual $\gamma_{k}$ is less than this α-quantile, i.e., $\gamma_{k}<\chi_{\alpha ,m}^{2}$, the standard cubature Kalman filter is carried out. Otherwise, if $\gamma_{k}\geq \chi_{\alpha ,m}^{2}$, it can be conclude with high probability (1−α) that there exists process uncertainty in the dynamic system. In this case, the improved strong tracking cubature Kalman filter is used.

#### 3.1.2. Improved Strategy for Fading Factor

In order to make different channel have different fading rate and respective filter adjustment capability in the multi-dimensional GPS/INS integrated navigation systems, a defined multiple time-varing suboptimal fading factor matrix $\Lambda_{k}$ to the prediction error covariance matrix is introduced:

(31) $$P_{k|k-1}=\Lambda_{k}F_{k|k-1}P_{k-1}F_{k|k-1}^{T}+Q_{k-1}$$

where $\Lambda_{k}=diag\left\{1,1,1,\lambda_{4,k},\lambda_{5,k},\ldots ,\lambda_{9,k},\ldots 1,\ldots 1\right\}$ is the matrix of multiple time-varying suboptimal fading factor. Specifically, $\left\{\sqrt{\lambda_{4,k}},\sqrt{\lambda_{5,k}},\ldots ,\sqrt{\lambda_{9,k}}\right\}$ is used to adjust the state variable that can be directly observed in the GPS/INS integrated navigation system which includes the velocity error and position error, whereas the other elements of $\Lambda_{k}$ is set as 1 since they cannot be estimated adaptively due to their unobservablities. The defined multiple time-varing fading factor $\Lambda_{k}$ can be determined as follows:

According to the orthogonality principle [31], in order to satisfy $E\left[\varepsilon_{k+j}\varepsilon_{k}^{T}\right]=0$, we need to make the following equation hold:

(32) $$P_{k|k-1}H_{k}^{T}-K_{k}V_{k}\equiv 0$$

Substituting (4) into (32) yields:

(33) $$P_{k|k-1}H_{k}^{T}-P_{k|k-1}H_{k}^{T}{\left(H_{k}P_{k|k-1}H_{k}^{T}+R_{k}\right)}^{-1}V_{k}\equiv 0$$

Simplifying Equation (33), we have:

(34) $$H_{k}P_{k|k-1}H_{k}^{T}=V_{k}-R_{k}$$

Substituting (31) and the fading factor $\Lambda_{k}$ into (34) yields:

(35) $$H_{k}\Lambda_{k}F_{k|k-1}P_{k-1}F_{k|k-1}^{T}H_{k}^{T}=V_{k}-H_{k}Q_{k-1}H_{k}^{T}-R_{k}$$

To be specific, for the GPS/INS integrated navigation system with the integration mode of velocity and position, the observation matrix can be expressed as:

(36) $$H_{k}=\left[\begin{array}{ccc} 0_{6\times 3} & I_{6\times 6} & 0_{6\times 6} \end{array}\right]$$

Here we define:

(37) $$\Delta_{k}=H_{k}\Lambda_{k}=\left[\begin{array}{ccc} 0_{6\times 3} & \begin{array}{cccccc} \lambda_{4,k} & 0 & 0 & 0 & 0 & 0\\ 0 & \lambda_{5,k} & 0 & 0 & 0 & 0\\ 0 & 0 & \lambda_{6,k} & 0 & 0 & 0\\ 0 & 0 & 0 & \lambda_{7,k} & 0 & 0\\ 0 & 0 & 0 & 0 & \lambda_{8,k} & 0\\ 0 & 0 & 0 & 0 & 0 & \lambda_{9,k} \end{array} & 0_{6\times 6} \end{array}\right]$$

(38) $$G_{k}=F_{k|k-1}P_{k-1}F_{k|k-1}^{T}H_{k}^{T}$$

Substituting (37) and (38) into (35) yields:

(39) $$\Delta_{k}G_{k}=N_{k}$$

i.e.,:

(40) $$\left[\begin{array}{ccc} 0_{6\times 3} & \begin{array}{cccccc} \lambda_{4,k} & 0 & 0 & 0 & 0 & 0\\ 0 & \lambda_{5,k} & 0 & 0 & 0 & 0\\ 0 & 0 & \lambda_{6,k} & 0 & 0 & 0\\ 0 & 0 & 0 & \lambda_{7,k} & 0 & 0\\ 0 & 0 & 0 & 0 & \lambda_{8,k} & 0\\ 0 & 0 & 0 & 0 & 0 & \lambda_{9,k} \end{array} & 0_{6\times 6} \end{array}\right]G_{k}=N_{k}$$

Using the method of least squares we have:

(41) $$\lambda_{4,k}=\frac{\sum_{j=1}^{6}G_{k}\left[4,j\right]N_{k}\left[1,j\right]}{\sum_{j=1}^{6}G_{k}^{2}\left[4,j\right]}$$

Similarly, we obtain the $\lambda_{5,k}∼\lambda_{9,k}$. Then the multiple suboptimal fading factor $\Lambda_{k}$ for the low-cost GPS/INS integration navigation system can be estimated adaptively as:

(42) $$\Lambda_{i,k}=\left\{ \begin{array}{ll} \frac{\sum_{j=1}^{6}G_{k}\left[i,j\right]N_{k}\left[i-3,j\right]}{\sum_{j=1}^{6}G_{k}^{2}\left[i,j\right]} & ,i=4,5,\cdots ,9\\ 1 & \;,\;i=1,2,3,10,\cdots ,21 \end{array}\right.$$

Unfortunately, the symmetry of the prediction covariance matrix $P_{k|k-1}$ in (31) cannot be guaranteed when the suboptimal fading factor $\Lambda_{k}$ with different diagonal elements, i.e., $\lambda_{4,k}\neq \lambda_{5,k}\neq \ldots \neq \lambda_{9,k}$, is carried out on the matrix $F_{k|k-1}P_{k}F_{k|k-1}^{T}$. To solve the problem, the square root filter method is considered. Based on the idea of Cholesky decomposition, the fading factor matrix $\Lambda_{k}$ can be decomposed into:

(43) $$\Lambda_{k}={\hat{\Lambda }}_{k}\cdot {\hat{\Lambda }}_{k}^{T}$$

where ${\hat{\Lambda }}_{k}=diag\left\{1,1,1,\sqrt{\lambda_{4,k}},\sqrt{\lambda_{5,k}},\ldots ,\sqrt{\lambda_{9,k}},\ldots 1,\ldots 1\right\}$. Hence, by introducing the improved two multiple fading factor ${\hat{\Lambda }}_{k}$, the prediction error covariance matrix can be rewritten as:

(44) $$P_{k|k-1}={\hat{\Lambda }}_{k}F_{k|k-1}P_{k}F_{k|k-1}^{T}{\hat{\Lambda }}_{k}{}^{T}+Q_{k-1}$$

By using Equation (44), the properties of the prediction error covariance matrix such as symmetric positive definite can be ensured and the stability of the filter can be therefore enhanced.

### 3.2. The Seventh-Degree Spherical Simplex-Radial Cubature Rule for Cubature Kalman Filter

The cubature Kalman filter based on the third-degree spherical-radial cubature rule has been successfully applied in many practical applications. However, it still has limited estimation accuracy. For example, the cubature Kalman filter can’t calculate exactly the Gaussian weighted integral of such simple polynomial functions as $x_{1}^{2}x_{2}^{2}$, where $x_{1}$ and $x_{2}$ are the two components of the Gaussian random vector function [33]. In order to overcome the shortcoming and further improve the accuracy and efficiency of the traditional CKF, a high-degree cubature Kalman filter based on the seventh-degree spherical simplex-radial rule is developed in this section.

In the seventh-degree spherical simplex-radial cubature rules, the following integral is considered:

(45) $$I\left(f\right)=\int_{{\mathbb{R}}^{n}}f(x)\;\exp(-x^{T}x)dx$$

where f(x) is some arbitrary nonlinear function, ${\mathbb{R}}^{n}$ is the domain of integration, and $\exp(-x^{T}x)$ is the weighting function. Let x=rs with $s^{T}s=1$ and $r=\sqrt{x^{T}x}$, Equation (45) can be rewritten in the spherical-radial coordinate system as:

(46) $$I\left(f\right)=\int_{0}^{\infty }\int_{U_{n}}f(rs)r^{n-1}\exp(-r^{2})d\sigma (s)dr$$

where $U_{n}=\left\{s\in {\mathbb{R}}^{n}|s^{T}s=1\right\}$ is the spherical surface and σ(⋅) is the spherical surface measure or the area element on $U_{n}$. Then, the Equation (46) can be decomposed into the spherical integral $S(r)=\int_{U_{n}}f(rs)d\sigma (s)$ with the weighting function $\omega_{f}(s)=1$, and the radial integral $\int_{0}^{\infty }S(r)r^{n-1}\exp(-r^{2})dr$ with the weighing function $\omega_{f}(r)=r^{n-1}\exp(-r^{2})$.

#### 3.2.1. Seventh-Degree Spherical Simplex Rule

The spherical integral $S(r)=\int_{U_{n}}f(rs)d\sigma (s)$ can be derived based on the transformation group of the regular simplex, with vertices $a_{j}={\left[a_{j,1},a_{j,2},\cdots ,a_{j,n}\right]}^{T},j=1,2,\cdots ,n+1$ as follows [34]:

(47) $$a_{j,i}\equiv \{\begin{array}{ll} 0, & i>j\\ \sqrt{\frac{\left(n+1\right)\left(n-j+1\right)}{n\left(n-j+2\right)}}, & i=j\\ -\sqrt{\frac{n+1}{n\left(n-i+2\right)\left(n-i+1\right)}}, & i<j \end{array}$$

For example, when the dimension *n* = 2, we can obtain that $\left\{a_{j}\right\}=\left\{{\left[1,0\right]}^{T},{\left[-1/2,\sqrt{3}/2\right]}^{T},{\left[-1/2,-\sqrt{3}/2\right]}^{T}\right\}$. Based on the projection from the midpoints of the vector $a_{j}$, we can obtain the following point sets [34]:

(48) $$\left\{b_{j}\right\}\equiv \left\{\sqrt{\frac{n}{2\left(n-1\right)}}\left(a_{i}+a_{j}\right):i<j\right\}$$

(49) $$\left\{c_{j}\right\}\equiv \left\{\sqrt{\frac{n}{3\left(n-2\right)}}\left(a_{i}+a_{j}+a_{m}\right):i<j<m,m=1,2,\ldots ,n+1\right\}$$

(50) $$\left\{d_{j}\right\}\equiv \left\{\sqrt{\frac{n}{10n-6}}\left(a_{i}+3a_{j}\right):i\neq j\right\}$$

where $\left\{b_{j}\right\}$ are the set of midpoints of the vertices $a_{j}$ projected onto the surface of the sphere $U_{n}$, $\left\{c_{j}\right\}$ are projections of the centroid of vertices $a_{j}$ faces onto $U_{n}$, and $\left\{d_{j}\right\}$ are projections of the selected edge points of the vertices $a_{j}$ onto the surface of $U_{n}$.

Taking the sets $\left\{a_{j}\right\}$, $\left\{b_{j}\right\}$, $\left\{c_{j}\right\}$ and $\left\{d_{j}\right\}$ as cubature points and further employing the central symmetry of the cubature formula, we can obtain the seventh-degree spherical simplex rule with $\left(n+1\right)\left(n^{2}+8n+6\right)/3$ cubature points as follows [34,35]:

(51) $$\begin{array}{ll} S_{7}\left(r\right) & =\frac{\left|A_{n}\right|}{36n{\left(n+1\right)}^{3}\left(n+2\right)\left(n+4\right)}\{n^{3}\left(9n^{2}-793n+1800\right)\sum_{j=1}^{n+1}\left[f\left(ra_{j}\right)+f\left(-ra_{j}\right)\right]\\ & +144{\left(n-1\right)}^{3}\left(4-n\right)\sum_{j=1}^{n\left(n+1\right)/2}\left[f\left(rb_{j}\right)+f\left(-rb_{j}\right)\right]\\ & +486{\left(n-2\right)}^{3}\sum_{j=1}^{\left(n-1\right)n\left(n+1\right)/6}\left[f\left(rc_{j}\right)+f\left(-rc_{j}\right)\right]\\ & +{\left(10n-6\right)}^{3}\sum_{j=1}^{n\left(n+1\right)}\left[f\left(rd_{j}\right)+f\left(-rd_{j}\right)\right]\} \end{array}$$

where $A_{n}=2\Gamma \left(\frac{1}{2}\right)/\Gamma \left(\frac{n}{2}\right)=2\sqrt{\pi^{n}}/\Gamma \left(\frac{n}{2}\right)$ is the surface area of the unit sphere, the Gamma function is defined as $\Gamma \left(n\right)=\int_{0}^{\infty }x^{n-1}e^{-x}dx$ with the properties of $\Gamma \left(1/2\right)=\sqrt{\pi }$ and Γ(n+1)=nΓ(n).

#### 3.2.2. Seven-Degree Radial Rule

Based on the idea of moment matching, the radial integral can be calculated by the following moment equation:

(52) $$\int_{0}^{\infty }S\left(r\right)r^{n-1}e^{-r^{2}}dr\approx \sum_{i=1}^{N_{r}}w_{r,i}S\left(r_{i}\right)$$

where $r_{i}$ and $w_{r,i}$ denote the quadrature point and the weights, respectively. $N_{r}$ is the total number of points; $S\left(r\right)=r^{l}$ is a monomial in r, with l an even integer. Then, the left hand side of the Equation (52) is reduced to $\frac{1}{2}\Gamma \left(\frac{n+l}{2}\right)$ with $\Gamma \left(n\right)=\int_{0}^{\infty }x^{n-1}e^{-x}dx$, i.e.,:

(53) $$\int_{0}^{\infty }r^{l}r^{n-1}e^{-r^{2}}dr=\frac{1}{2}\Gamma \left(\frac{n+l}{2}\right)$$

Because the spherical rule and the resultant spherical-radial cubature rule are fully symmetric, we only need to match the even-degree monomials. The quadrature point $r_{i}$ and $w_{r,i}$ can therefore be obtained by solving the different even-degree monomials matching. For the seventh-degree radial rule, we require that the points and weights satisfy the following four equations:

(54) $$\left\{ \begin{array}{l} w_{r,1}r_{1}^{0}+w_{r,2}r_{2}^{0}=\frac{1}{2}\Gamma \left(\frac{n}{2}\right)\\ w_{r,1}r_{1}^{2}+w_{r,2}r_{2}^{2}=\frac{1}{2}\Gamma \left(\frac{n}{2}+1\right)=\frac{n}{4}\Gamma \left(\frac{n}{2}\right)\\ w_{r,1}r_{1}^{4}+w_{r,2}r_{2}^{4}=\frac{1}{2}\Gamma \left(\frac{n}{2}+2\right)=\frac{1}{2}\left(\frac{n}{2}+1\right)\left(\frac{n}{2}\right)\Gamma \left(\frac{n}{2}\right)\\ w_{r,1}r_{1}^{6}+w_{r,2}r_{2}^{6}=\frac{1}{2}\Gamma \left(\frac{n}{2}+3\right)=\frac{1}{2}\left(\frac{n}{2}+2\right)\left(\frac{n}{2}+1\right)\left(\frac{n}{2}\right)\Gamma \left(\frac{n}{2}\right) \end{array}\right.$$

By solving the above four equations of zeroth, second, fourth, and sixth order moments, the points and weights for the seventh-degree radial rule with 2 quadrature points can be obtained as follows:

(55) $$\left\{ \begin{array}{l} r_{1}=\frac{1}{2}\sqrt{4+2n+2\sqrt{4+2n}}\\ r_{2}=\frac{1}{2}\sqrt{4+2n-2\sqrt{4+2n}} \end{array}\right.$$

(56) $$\left\{ \begin{array}{l} w_{r,1}=\left(\frac{1}{4}-\frac{1}{2\sqrt{4+2n}}\right)\Gamma \left(\frac{n}{2}\right)\\ w_{r,2}=\left(\frac{1}{4}+\frac{1}{2\sqrt{4+2n}}\right)\Gamma \left(\frac{n}{2}\right) \end{array}\right.$$

A detailed calculation of the points and weights can be found in [33]. Then, the seventh-degree radial rule can be obtained as follows:

(57) $$\int_{0}^{\infty }S\left(r\right)r^{n-1}e^{-r^{2}}dr=w_{r,1}S\left(r_{1}\right)+w_{r,2}S\left(r_{2}\right)$$

where $w_{r,1}$, $r_{1}$, $w_{r,2}$, $r_{2}$ are given by Equations (55) and (56).

#### 3.2.3. Seventh-Degree Spherical Simplex-Radial Rule

Combining Equations (51) and (57), the seventh-degree Spherical Simplex-Radial (SSR) rule can be formulated as:

(58) $$\begin{array}{l} \int_{{\mathbb{R}}^{n}}f\left(x\right)N\left(x;\bar{x},P_{x}\right)dx=\left(\frac{1}{2}-\frac{1}{\sqrt{4+2n}}\right)\frac{n^{2}\left(9n^{2}-793n+1800\right)}{36{\left(n+1\right)}^{3}\left(n+2\right)\left(n+4\right)}\sum_{j=1}^{n+1}[f\left(\bar{x}-\sqrt{\left(n+2+\sqrt{4+2n}\right)P_{x}}a_{j}\right)\\ +f\left(\bar{x}+\sqrt{\left(n+2+\sqrt{4+2n}\right)P_{x}}a_{j}\right)]+\left(\frac{1}{2}-\frac{1}{\sqrt{4+2n}}\right)\frac{4{\left(n-1\right)}^{3}\left(4-n\right)}{n{\left(n+1\right)}^{3}\left(n+2\right)\left(n+4\right)}\\ \sum_{k=1}^{n\left(n+1\right)/2}[f\left(\bar{x}-\sqrt{\left(n+2+\sqrt{4+2n}\right)P_{x}}b_{k}\right)+f\left(\bar{x}+\sqrt{\left(n+2+\sqrt{4+2n}\right)P_{x}}b_{k}\right)]\\ +\left(\frac{1}{2}-\frac{1}{\sqrt{4+2n}}\right)\frac{486{\left(n-2\right)}^{3}}{36n{\left(n+1\right)}^{3}\left(n+2\right)\left(n+4\right)}\sum_{k=1}^{\left(n-1\right)n\left(n+1\right)/6}[f\left(\bar{x}-\sqrt{\left(n+2+\sqrt{4+2n}\right)P_{x}}c_{k}\right)\\ +f\left(\bar{x}+\sqrt{\left(n+2+\sqrt{4+2n}\right)P_{x}}c_{k}\right)]+\left(\frac{1}{2}-\frac{1}{\sqrt{4+2n}}\right)\frac{{\left(10n-6\right)}^{3}}{36n{\left(n+1\right)}^{3}\left(n+2\right)\left(n+4\right)}\\ \sum_{k=1}^{n\left(n+1\right)}[f\left(\bar{x}-\sqrt{\left(n+2+\sqrt{4+2n}\right)P_{x}}d_{k}\right)+f\left(\bar{x}+\sqrt{\left(n+2+\sqrt{4+2n}\right)P_{x}}d_{k}\right)] \end{array}$$

### 3.3. Steps of the IST-7thSSRCKF

Based on the basic framework of spherical simplex-radial CKF, the proposed IST-7thSSRCKF algorithm is constructed by incorporating the strong tracking filter (STF) with an improved fading factor into the time update equation of seventh-degree SSRCKF. Hence, both the robustness against the process uncertainty and high accuracy can be achieved. The proposed IST-7thSSRCKF involves four steps for an iteration: (1) The state prediction; (2) Process uncertainty identification and calculation of the improved fading factor; (3) Covariance prediction using the improved strong tracking technique; (4) The measurement update.

*Initialization*: initialize state vector ${\hat{x}}_{0|0}$ and state covariance matrix $P_{0|0}$

- Step 1. State prediction

Generate cubature points by employing the seventh-degree spherical simplex-radial cubature rule as follows:

(59) $$P_{k-1|k-1}=S_{k-1|k-1}S_{k-1|k-1}^{T}$$

(60) $$X_{i,k-1|k-1}=S_{k-1|k-1}\xi_{i}+{\hat{x}}_{k-1|k-1}$$

$$\xi_{i}=\left\{ \begin{array}{ll} \bar{x}+\sqrt{\left(n+2+\sqrt{4+2n}\right)P_{x}}a_{i} & i=1,\cdots ,n+1,\\ \bar{x}-\sqrt{\left(n+2+\sqrt{4+2n}\right)P_{x}}a_{i-n-1} & i=n+2,\cdots ,2n+2,\\ \bar{x}+\sqrt{\left(n+2+\sqrt{4+2n}\right)P_{x}}b_{i-2n-2} & i=2n+3,\cdots ,\left(n^{2}+5n+4\right)/2,\\ \bar{x}-\sqrt{\left(n+2+\sqrt{4+2n}\right)P_{x}}b_{i-\left(n^{2}+5n+4\right)/2} & i=\left(n^{2}+5n+6\right)/2,\cdots ,n^{2}+3n+2,\\ \bar{x}+\sqrt{\left(n+2+\sqrt{4+2n}\right)P_{x}}c_{i-n^{2}-3n-2} & i=n^{2}+3n+3,\cdots ,\left(n^{3}+6n^{2}+17n+12\right)/6,\\ \bar{x}-\sqrt{\left(n+2+\sqrt{4+2n}\right)P_{x}}c_{i-\left(n^{3}+6n^{2}+17n+12\right)/6} & i=\left(n^{3}+5n^{2}+18n+18\right)/6,\cdots ,\left(n^{3}+3n^{2}+8n+6\right)/3,\\ \bar{x}+\sqrt{\left(n+2+\sqrt{4+2n}\right)P_{x}}d_{i-\left(n^{3}+3n^{2}+8n+6\right)/3} & i=\left(n^{3}+3n^{2}+8n+9\right)/3,\cdots ,\left(n^{3}+6n^{2}+11n+6\right)/3,\\ \bar{x}-\sqrt{\left(n+2+\sqrt{4+2n}\right)P_{x}}d_{i-\left(n^{3}+6n^{2}+11n+6\right)/3} & i=\left(n^{3}+6n^{2}+11n+9\right)/3,,\cdots ,\left(n^{3}+9n^{2}+14n+6\right)/3. \end{array}\right.$$

where *n* denotes the dimension of state vector $x_{k}$; the points sets $\left\{a_{i}\right\}$, $\left\{b_{i}\right\}$, $\left\{c_{i}\right\}$ and $\left\{d_{i}\right\}$ can be obtained by Equations (47)–(50).

Propagate the cubature points through the nonlinear state equation, the predicted state ${\hat{x}}_{k|k-1}$ and predicted error covariance without the fading factor $P_{k|k-1}^{l}$ can be given by:

(61) $$\chi_{i,k|k-1}^{*}=f\left(\chi_{i,k-1}\right)$$

(62) $${\hat{x}}_{k|k-1}=\sum_{i=1}^{\left(n^{3}+9n^{2}+14n+6\right)/3}\omega_{i}\chi_{i,k|k-1}^{*}$$

(63) $$P_{k|k-1}^{l}=\sum_{i=1}^{\left(n^{3}+9n^{2}+14n+6\right)/3}\omega_{i}\left(\chi_{i,k|k-1}^{*}-{\hat{x}}_{k|k-1}\right){\left(\chi_{i,k|k-1}^{*}-{\hat{x}}_{k|k-1}\right)}^{T}+Q_{k-1}$$

where $X_{i,k|k-1}^{*}$ denote the propagated cubature points, $Q_{k-1}$ denotes the covariance matrix of process noise, $\omega_{i}$ represent the corresponding weights of the cubature points and can be obtained by:

$$\omega_{i}=\left\{ \begin{array}{ll} \left(\frac{1}{2}-\frac{1}{\sqrt{4+2n}}\right)\frac{n^{2}\left(9n^{2}-793n+1800\right)}{36{\left(n+1\right)}^{3}\left(n+2\right)\left(n+4\right)} & i=1,\cdots ,2n+2,\\ \left(\frac{1}{2}-\frac{1}{\sqrt{4+2n}}\right)\frac{4{\left(n-1\right)}^{3}\left(4-n\right)}{n{\left(n+1\right)}^{3}\left(n+2\right)\left(n+4\right)} & i=2n+3,\cdots ,n^{2}+3n+2,\\ \left(\frac{1}{2}-\frac{1}{\sqrt{4+2n}}\right)\frac{486{\left(n-2\right)}^{3}}{36n{\left(n+1\right)}^{3}\left(n+2\right)\left(n+4\right)} & i=n^{2}+3n+3,\cdots ,\left(n^{3}+3n^{2}+8n+6\right)/3,\\ \left(\frac{1}{2}-\frac{1}{\sqrt{4+2n}}\right)\frac{{\left(10n-6\right)}^{3}}{36n{\left(n+1\right)}^{3}\left(n+2\right)\left(n+4\right)} & i=\left(n^{3}+3n^{2}+8n+12\right)/6,\cdots ,\left(n^{3}+9n^{2}+14n+6\right)/3. \end{array}\right.$$

- Step 2. Process uncertainty identification and calculation of the improved fading factor

Calculate the innovation $\varepsilon_{k}$ according to Equation (29) and identify the process uncertainty by using the hypothesis testing method described in:

(a) If $\varepsilon_{k}<\chi_{\alpha ,m}^{2}$, $\Lambda_{k}=diag\left\{1,1,1,\ldots 1,\ldots 1\right\}$, the strong tracking cubature Kalman filter therefore reduces to the standard cubature Kalman filter.
(b) If $\varepsilon_{k}\geq \chi_{\alpha ,m}^{2}$, perform the improved strong tracking cubature Kalman filter. The improved multiple fading factor used in the IST-7thSSRCKF is calculated as follows:
  (64) $$\Lambda_{i,k}=\left\{ \begin{array}{ll} \frac{\sum_{j=1}^{6}G_{k}\left[i,j\right]N_{k}\left[i-3,j\right]}{\sum_{j=1}^{6}G_{k}^{2}\left[i,j\right]} & ,i=4,5,\cdots ,9\\ 1 & ,\;i=1,2,3,10,\cdots ,21 \end{array}\right.$$

- Step 3. Covariance prediction using the improved strong tracking technique

Then, by introducing the improved multiple fading factor according to Equation (64), the modified prediction covariance can be calculated as:

(65) $$P_{k|k-1}=\sqrt{\Lambda_{i,k}}\left(P_{k|k-1}^{l}-Q_{k-1}\right){\sqrt{\Lambda_{i,k}}}^{T}+Q_{k-1}$$

- Step 4. Measurement update

By utilizing the predicted state ${\hat{x}}_{k|k-1}$ and the modified predicted covariance $P_{k|k-1}$ in (65), we can obtain the modified predicted measurement ${\hat{z}}_{k|k-1}$, the innovation covariance matrix $P_{zz,k|k-1}$ and the cross covariance matrix $P_{xz,k|k-1}$ as follows:

(66) $$X_{i,k|k-1}^{}=chol\left(P_{k|k-1}\right)\xi_{i}+{\hat{x}}_{k|k-1}$$

(67) $$Z_{i,k|k-1}=h\left(X_{i,k|k-1}\right)$$

(68) $${\hat{z}}_{k|k-1}=\sum_{i=1}^{\left(n^{3}+9n^{2}+14n+6\right)/3}\omega_{i}Z_{i,k|k-1}$$

(69) $$P_{zz,k|k-1}=\sum_{i=1}^{\left(n^{3}+9n^{2}+14n+6\right)/3}\omega_{i}\left(Z_{i,k|k-1}-{\hat{z}}_{k|k-1}\right){\left(Z_{i,k|k-1}-{\hat{z}}_{k|k-1}\right)}^{T}+R_{k}$$

(70) $$P_{xz,k|k-1}=\sum_{i=1}^{\left(n^{3}+9n^{2}+14n+6\right)/3}\omega_{i}\left(X_{i,k|k-1}-{\hat{x}}_{k|k-1}\right){\left(Z_{i,k|k-1}-{\hat{z}}_{k|k-1}\right)}^{T}$$

Finally, the state estimate ${\hat{x}}_{k|k}$ and the corresponding covariance $P_{k|k}$ at time k are calculated by Equations (26)–(28).

## 4. Performance Evaluation

In this section, the simulation and experimental results are presented to validate the proposed method. First, a new nonlinear filtering model that is suitable for the low-cost GPS/INS integrated navigation system is designed. Second, simulations and car-mounted experiments are carried out to evaluate the performance of different filters.

### 4.1. Design of SINS/GPS Filtering Model

In this paper, a 21-dimension state vector is adopted in the GPS/INS loosely coupled navigation system, which is given by:

(71) $$x={\left[\psi \;\;\;\;\;\delta v\;\;\;\;\;\delta p\;\;\;\;b_{g}\;\;\;b_{f}\;\;\;\delta b_{g}\;\;\delta b_{f}\right]}^{T}$$

where $\psi \;=\left[\begin{array}{ccc} \psi_{N} & \psi_{U} & \psi_{E} \end{array}\right]$, $\delta v\;\;=\left[\begin{array}{ccc} \delta V_{N} & \delta V_{U} & \delta V_{E} \end{array}\right]$ and $\delta p=\left[\begin{array}{ccc} \delta \lambda & \delta L & \delta h \end{array}\right]$ are the attitude error vector, velocity error vector and position error vector respectively. $b_{g}\;$ denotes the static bias and $\delta b_{g}\;$ is the dynamic bias noise of gyroscope expressed in body frame. Similarity, $b_{f}$ and $\delta b_{f}$ are the static and dynamic bias of three axis accelerometer under the body frame respectively. The nonlinear system error model of INS is defined by:

(72) $$\left\{ \begin{array}{l} \dot{\psi }=C_{w}\left[\left(I-C_{n}^{p}\right)w_{in}^{n}+\delta w_{in}^{n}-C_{b}^{p}\delta w_{ib}^{b}\right]\\ \delta \dot{v}=\left(I-C_{p}^{n}\right)C_{b}^{p}f_{ib}^{b}+C_{b}^{p}\delta f_{ib}^{b}+\delta v^{n}\times \left(2w_{ie}^{n}+w_{en}^{n}\right)+v^{n}\times \left(2\delta w_{ie}^{n}+\delta w_{en}^{n}\right)\\ \delta \dot{\lambda }=\frac{\delta V_{E}}{R_{N}+h}\sec L+\delta L\frac{V_{E}\mathrm{secL}}{R_{N}+h}\tan L\\ \delta \dot{L}=\frac{\delta V_{N}}{R_{N}+h}\\ \delta \dot{h}=\delta V_{U}\\ {\dot{b}}_{g}=0\\ {\dot{b}}_{f}=0\\ \delta {\dot{b}}_{g}=-\frac{1}{\tau_{g}}\delta b_{g}+\eta_{g}\\ \delta {\dot{b}}_{f}=-\frac{1}{\tau_{f}}\delta b_{f}+\eta_{f} \end{array}\right.$$

where $C_{w}$ is the transformation matrix from angle rate to Euler angles, $C_{n}^{p}$ is the rotation matrix from *n*-frame(ideal navigation frame) to *p*-frame(actual navigation frame), $C_{b}^{p}$ is the rotation matrix from *b*-frame(body frame) to *p*-frame. $f_{ib}^{b}$ denotes the specific force expressed in body frame, $\delta f_{ib}^{b}$ is the corresponding error vector. $\omega_{ba}^{c}$ represents the rotation velocity of *a*-frame with respected to *b*-frame expressed in *c*-frame and $\delta \omega_{ba}^{c}$ are the corresponding error vector. ψ is the misalignment angle and $\begin{array}{ccc} \psi_{N} & \psi_{U} & \psi_{E} \end{array}$ are the component in north, up and east direction. $V_{E}$, $V_{N}$, $V_{U}$ are the velocity component in east, north and up direction, L, λ, h are the latitude, longitude, and height, and $\delta V_{E}$, $\delta V_{N}$, $\delta V_{U}$, δL, δλ, δh are the corresponding error. $R_{N}$ is the normal radius. $\tau_{g}$ and $\tau_{f}$ represent the correlation time of 1st-order Markovian process for gyroscope and accelerometer. $\eta_{g}$ and $\eta_{f}$ are the zero-mean Gaussian white noise process.

### 4.2. Simulation for SINS/GPS Integration

In this section, the proposed method is compared with the CKF [30], STCKF [25] and STSSRCKF [26] in the GPS/INS integrated navigation application. This application has been widely used as a benchmark to validate the performance of nonlinear filter due to its practical application value. The nonlinear model described in (72) will be employed as the dynamic process model, which can be given by:

(73) $$x_{k}=f\left(x_{k-1}\right)+v_{k-1}$$

where f(.) is the nonlinear dynamic model function in Equation (72), $v_{k-1}$ is Gaussian noise with zero mean and covariance $Q_{k}$.$Q_{k}$ is determined from the inertial sensor’s stochastic error and set as $Q_{k}$ = diag([0.2 $\deg/\sqrt{h}$, 0.2 $\deg/\sqrt{h}$, 0.2 $\deg/\sqrt{h}$, 0.003 $m/s^{2}/\sqrt{Hz}$, 0.003 $m/s^{2}/\sqrt{Hz}$, 0.003 $m/s^{2}/\sqrt{Hz}$, 0.2 $\deg/\sqrt{h}$, 0.2 $\deg/\sqrt{h}$, 0.2 $\deg/\sqrt{h}$, 0.003 $m/s^{2}/\sqrt{Hz}$, 0.003 $m/s^{2}/\sqrt{Hz}$, 0.003 $m/s^{2}/\sqrt{Hz}$])^2^. In order to show how the filters perform under process uncertainty, the process noise are generated according to [36]:

(74) $$v_{k}∼\{\begin{array}{ll} N\left(0,\;Q_{k}\right) & w.p.0.8\\ N\left(0,\;100Q_{k}\right) & w.p.0.2 \end{array}$$

where w.p. denotes “with probability”, i.e., eighty percent of process noise are generated from Gaussian distribution with nominal covariance $Q_{k}$, and twenty percent of process noise are drawn from Gaussian distribution with severely increased covariance.

The measurement model can be formulated as follows:

(75) $$z_{k}=H_{k}x_{k}+\omega_{k-1}$$

where $z_{k}=\left(\begin{array}{c} V_{gps}^{N}-V_{ins}^{N}\\ V_{gps}^{U}-V_{ins}^{U}\\ V_{gps}^{E}-V_{ins}^{E}\\ L_{gps}-L_{ins}\\ h_{gps}-h_{ins}\\ \lambda_{gps}-\lambda_{ins} \end{array}\right)$, the subscripts (*GPS* and *ins*) represent the velocity and geographical position obtained from GPS and INS, respectively. $H_{k}=\left[0_{6\times 3},I_{6\times 6},0_{6\times 12}\right]$ is the observation matrix. $\omega_{k-1}$ is the white Gaussian measurement noise with zero mean and covariance $R_{k}$ = diag([0.05 m/s, 0.05 m/s, 0.05 m/s, 1 m, 3 m, 1 m])^2^, which is determined from the GPS’s stochastic measurement error.

**Figure 1:**
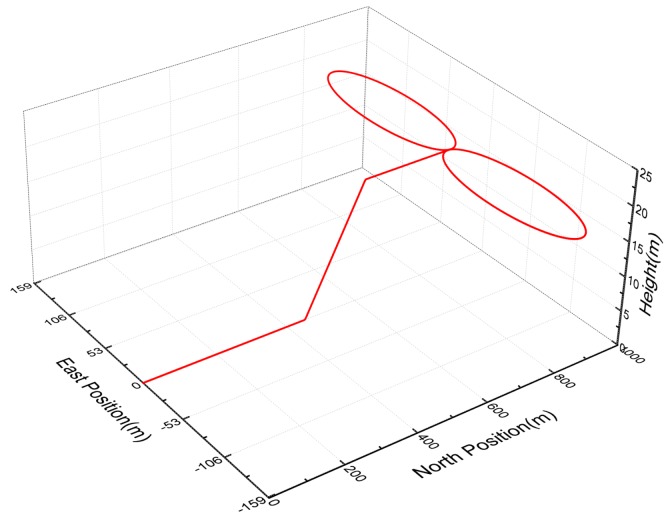
The three dimensional trajectory of the vehicle.

The simulation scenario is described in Table 1. For the first 30 s, the vehicle accelerates from 0 m/s to 30 m/s at the constant acceleration of 1 $m/s^{2}$, and then head up and decelerates from 30 m/s to 24 m/s at the constant acceleration of −1 $m/s^{2}$. Finally, the vehicle keeps this constant velocity and conducts the 8-driving. The three dimensional trajectory of the vehicle is shown in Figure 1.

In this simulation, the sample rate of IMU and GPS are 1000 Hz and 10 Hz, respectively. The output rate of GPS/SINS integration navigation system is 10 Hz. The initial state vector and the associated covariance are set as ${\hat{x}}_{0|0}$ = $0_{1\times 21}$, and **P**_0|0_ = diag([0.005 rad, 0.015 rad, 0.005 rad, 0.5 m/s, 0.5 m/s, 0.5 m/s, 10 m, 30 m, 10 m, 0.2 deg/s, 0.2 deg/s, 0.2 deg/s, 10 mg, 10 mg, 10 mg, 0.2 deg/s, 0.2 deg/s, 0.2 deg/s, 10 mg, 10 mg, 10 mg])^2^, respectively.

**Figure 2:**
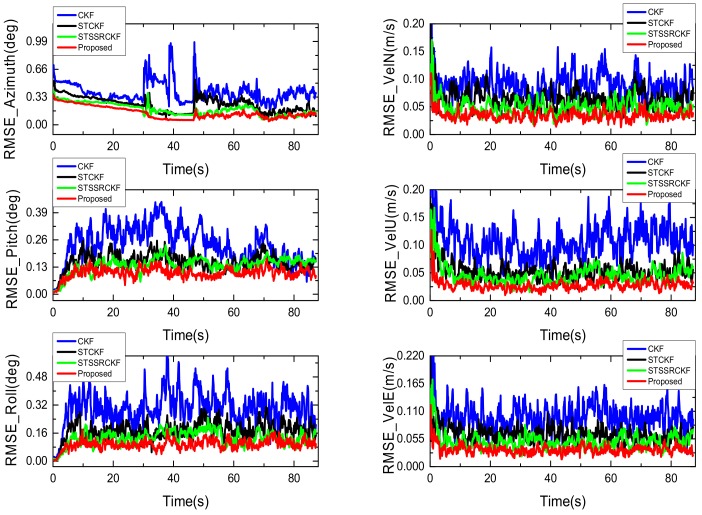
RMSEs of the attitude (**left**) and velocity (**right**).

Similar to [30], we conduct 50 independent Monte Carlo runs for fair comparison and use the root mean square error(RMSE) as the metrics to compare the performance of four filters. The RMSEs of the attitude and velocity obtained by these filters are shown in Figure 2, respectively.

As can be seen from Figure 2, both the RMSEs from the proposed IST-7thSSRCKF, STSSRCKF and STCKF are smaller than that of the existing CKF. It means that the strong tracking-based filter can suppress the process uncertainty effectively. Moreover, we can also see from Figure 2 that the proposed IST-7thSSRCKF using the seventh-degree spherical simplex-radial cubature rule outperforms the STCKF and STSSRCKF as expected, which means that the estimation accuracy of the proposed IST-7thSSRCKF is further improved by the new Gaussian integral rule. Thus, the proposed method is more robust against process uncertainty for the GPS/INS integration navigation system as compared with the existing filters.

### 4.3. Car-Mounted Experiment for SINS/GPS Integration

**Figure 3:**
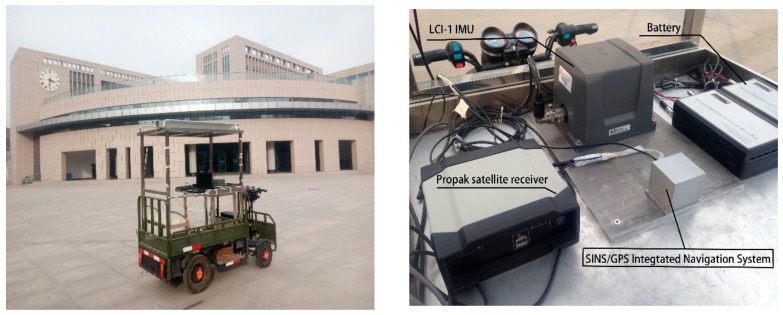
Setup of experimental platform.

**Figure 4:**
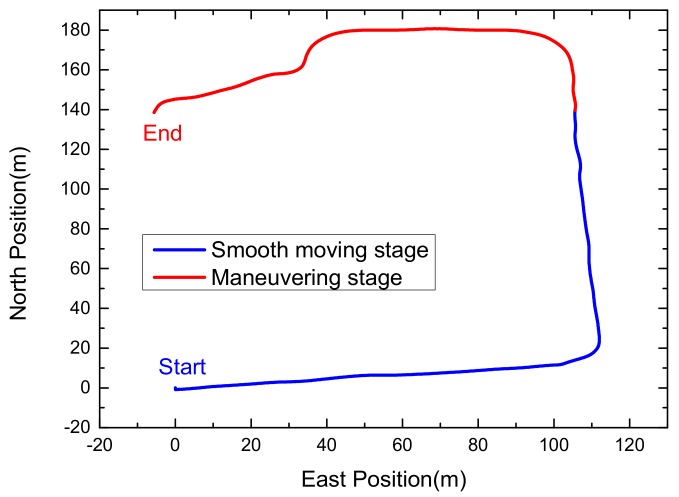
Car-mounted test trajectory.

**Figure 5:**
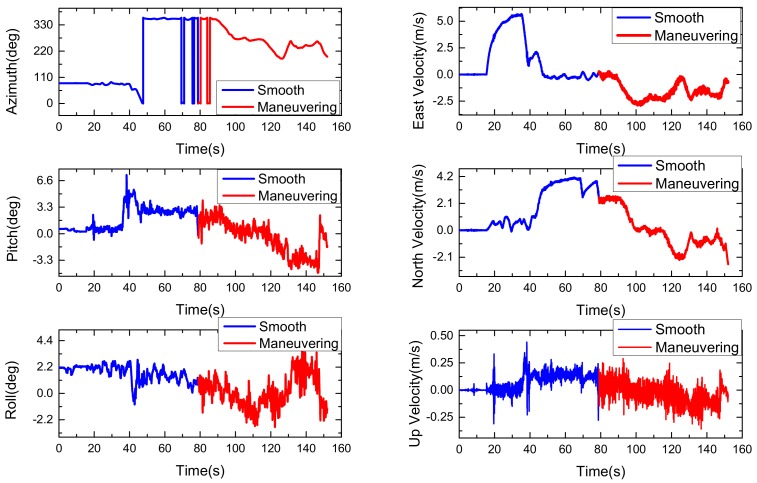
The reference attitude (**left**) and velocity (**right**) during the whole test.

**Figure 6:**
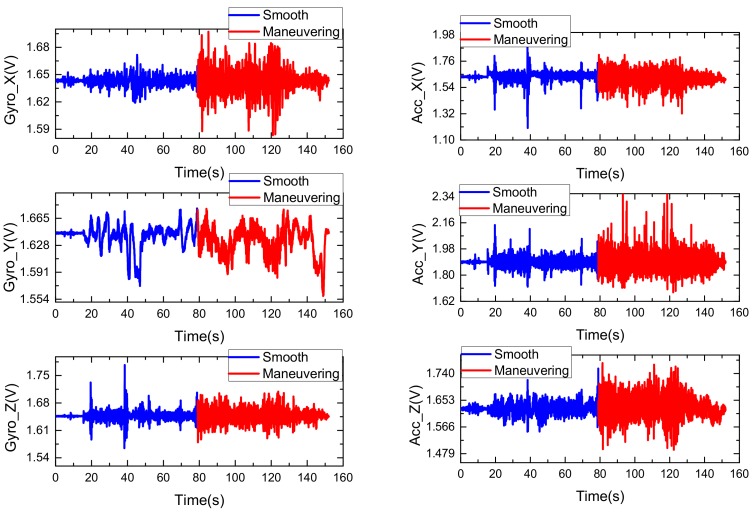
The original outputs of the tri-axis gyroscope (**left**) and accelerometer (**right**).

In order to verify the effectiveness and superiority of the proposed algorithm, a car-mounted experiment was carried out by the advanced navigation system research group of the North University of China (Taiyuan, China). As shown in Figure 3, the experimental platform is composed of a LCI-1 tactical grade inertial measurement unit (IMU) and a Propak satellite receiver, which uses Novatel synchronized position attitude navigation (SPAN) as the reference solution [37]. The attitude, velocity and position accuracy of the Novatel SPAN reference system are 0.01 deg, 0.05 m/s and 0.1 m, respectively. The sensors specification of our self-made MINS/GPS integration navigation system is listed in Table 2. The experiment is carried out in North University of China and the total test time is 152 s. In the experiment test, the car firstly moved smoothly from 0 s to 79 s and then maneuvered severely from 79 s to 152 s. The test trajectory is shown in Figure 4. The reference attitude, velocity and position provided by the high-precision integrated navigation system during the whole test are shown in Figure 5. The original output of the tri-axis gyroscope and accelerometer are shown in Figure 6. It can be clearly seen from Figure 6 that there are much stochastic process uncertainties during time interval 80–152 s.

### 4.4. Performance Comparison with Different Filtering Algorithms

In this section, the proposed filter is tested and compared with the existing CKF [30], strong tracking cubature Kalman filter (ST-CKF) [25] and strong tracing spherical simplex-radial cubature Kalman filter (STSSRCKF) [26] in the car-mounted experiment for the low-cost GPS/INS integration navigation. For all the four methods, the initial state and corresponding error covariance are chosen as ${\hat{x}}_{0|0}$ = [$0_{1\times 9}$, 0.2 deg/s, 0.2 deg/s, 0.2 deg/s, 16 mg, 16 mg, 16 mg, 0.0018 deg/s, 0.0018 deg/s, 0.0018 deg/s, 0.1 mg, 0.1 mg, 0.1 mg] and **P**_0|0_ = diag([0.005 rad, 0.015 rad, 0.005 rad, 0.1 m/s, 0.1 m/s, 0.1 m/s, 5 m, 10 m, 5 m, 0.2 deg/s, 0.2 deg/s, 0.2 deg/s, 16 mg, 16 mg, 16 mg, 0.0018 deg/s, 0.0018 deg/s, 0.0018 deg/s, 0.1 mg, 0.1 mg, 0.1 mg])^2^, respectively. The iSnitial value of the nominal process noise covariance matrix and measurement noise covariance matrix are respectively set as $Q_{k}$ = diag([0.3 $\deg/\sqrt{h}$, 0.3 $\deg/\sqrt{h}$, 0.3 $\deg/\sqrt{h}$, 0.3 $m/s^{2}/\sqrt{Hz}$, 0.3 $m/s^{2}/\sqrt{Hz}$, 0.3 $m/s^{2}/\sqrt{Hz}$, 0.3 $\deg/\sqrt{h}$, 0.3 $\deg/\sqrt{h}$, 0.3 $\deg/\sqrt{h}$, 0.3 $m/s^{2}/\sqrt{Hz}$, 0.3 $m/s^{2}/\sqrt{Hz}$, 0.3 $m/s^{2}/\sqrt{Hz}$ ])^2^ and $R_{k}$ = diag([0.1 m/s, 0.1 m/s, 0.1 m/s, 5 m, 10 m, 5 m])^2^. In the ST-CKF, the forgetting factor ρ and softening factor β are empirically selected as 0.95 and 4.5 for optimal purpose. In the proposed improved strong tracking seventh-degree cubature Kalman filter (IST-7thSSRCKF), the parameters are set as: $\alpha =0.05,\chi_{\alpha ,m}^{2}=12.592$. The proposed algorithm and existing algorithms are all carried out by Matlab 2016 on a computer with an Intel core i5-3320 CPU at 2.60 GHz, 4 GB memory, and Windows 7 operating system. For a fair comparison, we conduct M = 20 independent runs. All the filters use the same parameter in each run.

**Figure 7:**
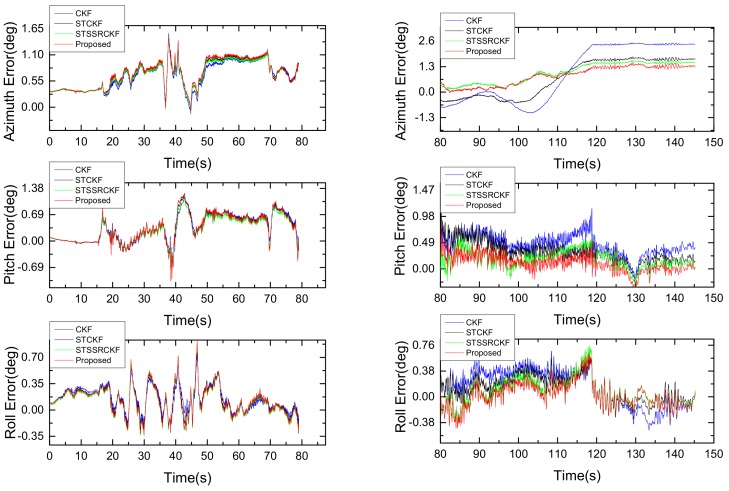
The attitude error in smooth stage (**left**) and maneuvering stage (**right**) from different filters.

**Figure 8:**
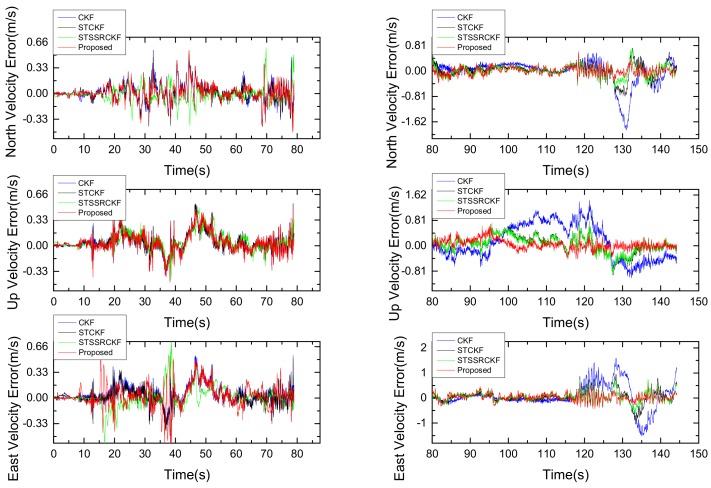
The velocity error in smooth stage (**left**) and maneuvering stage (**right**) from different filters.

The velocity and attitude error obtained from the four filtering algorithms when M = 10 are respectively shown in Figure 7 and Figure 8. Owing to the fact that the position error estimation is mainly determined by the measurement noise matrix R and is not sensitive to the time-varying process noise, it is unable to reflect the true performance of the four different filters in the maneuvering stage. For this reason, we do not present the result of the position error here. It can be seen from Figure 7 and Figure 8 that, in the smooth moving stage during intervals 0–79 s, the proposed method performs quite similar with the existing filters. The results are not surprising, this is because that in this case the Gaussian assumption for the statistical value of process noise can be satisfied. While for the maneuvering stage in the intervals of 80–152 s, the proposed IST-7thSSRCKF and the existing strong tracking based filters such as ST-CKF and ST-SSRCKF, exhibit smaller navigation error than the traditional CKF. This is because that in the maneuvering stage the Gaussian assumption of process noise is violated and the statistical value of process noise is changed due to the influence of the vehicle’s severe maneuvers, which results in a much degraded performance of CKF. In contrast, the severe vehicle maneuverability can be tracked appropriately by the ST-CKF, ST-SSRCKF and IST-7thSSRCKF as shown in Figure 7 and Figure 8. The performance improvement is mainly due to the strong tracking technique used in these filters, since the time-varying process uncertainty can be better suppressed by introducing the time-variant suboptimal fading factor matrix to the state prediction covariance matrix to maintain the residual sequence orthogonal. Furthermore, we can also see that the IST-7thSSRCKF outperforms the ST-CKF and ST-SSRCKF, which indicates that the 7th-degree SSR rule is more accurate in computing the multi-dimensional integrals than the SR and SSR rules. However, the accuracy improvement is at the cost of slightly larger computational burden as shown in Table 3. The reason is that the computation time is approximately proportional to the number of points used in these filters. The number of points required in the IST-7thSSRCKF is 4510, which is greater than that of CKF, STCKF and ST-SSRCKF, which are being 42, 42 and 44, respectively. In this respect, the implementation time of the proposed method is almost 100 times of CKF, STCKF and ST-SSRCKF. Actually, for the GPS/INS integrated navigation system with high performance navigation computer, the large computational burden can be afforded. Thereby, the proposed IST-7thSSRCKF is a better choice to deal with process uncertainty in terms of estimation accuracy when comparing with the investigated other three methods.

**Figure 9:**
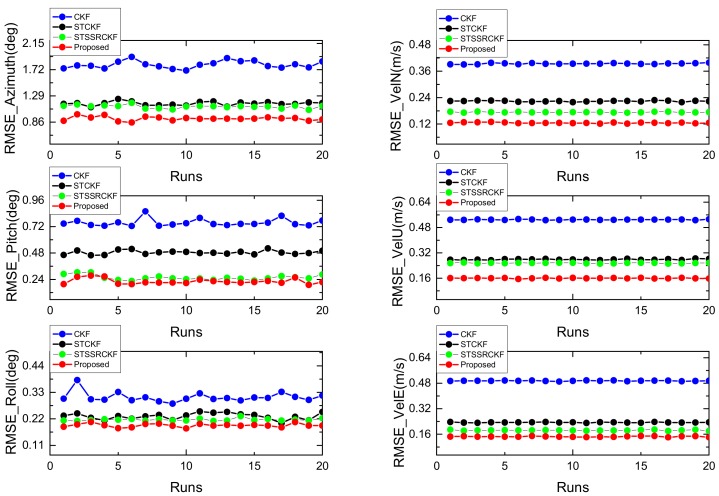
The RMSEs of attitude error (**left**) and velocity error (**right**) when M = 1:20.

In order to compare the overall performance of the four methods and intuitively illustrate the effectiveness of the proposed filter for low-cost GPS/INS integration, the root mean square error (RMSE) of the velocity and attitude that averaged across all time instances is used as a comparison metric, which are defined as follows:

$$RMSE=\sqrt{\frac{1}{N}\sum_{k=1}^{N}\left(x_{k}-{\hat{x}}_{k}\right)}$$

where *N* is the total sample number, $x_{k}$ and ${\hat{x}}_{k}$ denote the true value and the estimated value of velocity and attitude at time *k*, respectively. The RMSEs of the four methods in estimating the velocity and attitude of the low-cost GPS/INS integrated navigation system when M = 10 are listed in Table 4. The RMSEs of attitude error and velocity error when M = 1:20 are shown in Figure 9, respectively. It can be clearly seen from Table 4 and Figure 9 that the CKF exhibits large error in the maneuvering stage than the STCKF, STSSRCKF and IST-7thSSRCKF.

The STCKF and STSSRCKF maintain a good performance but are less accurate than the IST-7thSSRCKF. Therefore, taking both the estimation accuracy and computational efficiency into account, we can conclude that the proposed IST-7thSSRCKF can achieve high estimation accuracy with slightly higher computational complexity, and has better robustness for the suppression of process uncertainties.

## 5. Conclusions

In this paper, an improved strong tracking cubature Kalman filter is proposed to suppress the process uncertainty induced by the severe maneuvering for the low-cost GPS/INS integrated navigation systems. Based on the improved strong tracking technique, the process uncertainty can be detected and suppressed by modifying the prior state estimate covariance online according to the change in vehicle dynamics. Moreover, the seventh-degree spherical simplex-radial cubature rule, which can be used to compute the more exact Gaussian type integral, is integrated into the strong tracking cubature Kalman filter to further enhance the estimation accuracy. Thus, the proposed IST-7thSSRCKF possesses the advantage of 7thSSRCKF’s high accuracy and strong tracking filter’s strong robustness against process uncertainties. The car-mounted experiments are utilized to demonstrate that the proposed IST-7thSSRCKF can achieve high estimation accuracy and has better robustness for the suppression of process uncertainties.

## Figures and Tables

**Table 1:** Description of vehicle motion.

Time (s)	Motion
0–30	Accelerate
30–36	Head up and decelerate
37–47	Uniform
48–87	8-driving

**Table 2:** Specifications of the SINS.

Quantity	Gyroscope	Accelerometer
Range	±300°/s	±10 g
Bias	12°/h	5 mg
Random walk	0.28 $\deg/\sqrt{h}$	90 $\mathrm{ug}/\sqrt{Hz}$

**Table 3:** Number of points and computation complexity of different filter for each run.

Filers	Points Number	Time (s)
CKF	42	0.011
ST-CKF	42	0.016
ST-SSRCKF	44	0.018
IST-7thSSRCKF	4510	0.968

**Table 4:** RMSEs of different filters in the maneuvering stage when M = 10.

Filers	CKF	ST-CKF	ST-SSRCKF	IST-7thSSRCKF
Azimuth (deg)	1.70	1.19	1.11	0.96
Pitch (deg)	0.74	0.49	0.24	0.19
Roll (deg)	0.30	0.22	0.21	0.18
North Velocity (m/s)	0.39	0.22	0.17	0.12
Up Velocity (m/s)	0.50	0.27	0.25	0.16
East Velocity (m/s)	0.49	0.23	0.18	0.14
